# Suicide surveillance and health systems in Nepal: a qualitative and social network analysis

**DOI:** 10.1186/s13033-016-0073-7

**Published:** 2016-06-06

**Authors:** Ashley K. Hagaman, Uden Maharjan, Brandon A. Kohrt

**Affiliations:** School of Human Evolution and Social Change, Arizona State University, Tempe, USA; Health Research and Social Development Forum, Kathmandu, Nepal; Duke Global Health Institute and Department of Psychiatry and Behavioral Sciences, Duke University, Durham, NC USA

**Keywords:** Suicide, Vital surveillance, Health systems, Suicide detection, Nepal, Developing countries, Law enforcement, Policy

## Abstract

**Background:**

Despite increasing recognition of the high burden of suicide deaths in low- and middle-income countries, there is wide variability in the type and quality of data collected and reported for suspected suicide deaths. Suicide data are filtered through reporting systems shaped by social, cultural, legal, and medical institutions. Lack of systematic reporting may underestimate public health needs or contribute to misallocation of resources to groups most at risk.

**Methods:**

The goal of this study was to explore how institutional structures, cultural perspectives on suicide, and perceived criminality of self-harm influence the type and quality of suicide statistics, using Nepal as an example because of its purported high rate of suicide in the public health literature. Official documentation and reporting networks drawn by police, policy makers, and health officials were analyzed. Thirty-six stakeholders involved in various levels of the death reporting systems in Nepal participated in in-depth interviews and an innovative drawn surveillance system elicitation task.

**Results:**

Content analysis and social network analysis revealed large variation across the participants perceived networks, where some networks were linear pathways dominated by a single institution (police or community) with few nodes involved in data transmission, while others were complex and communicative. Network analysis demonstrated that police institutions controlled the majority of suicide information collection and reporting, whereas health and community institutions were only peripherally involved. Both health workers and policy makers reported that legal codes criminalizing suicide impaired documentation, reporting, and care provision. However, legal professionals and law review revealed that attempting suicide is not a crime punishable by incarceration. Another limitation of current reporting was the lack of attention to male suicide.

**Conclusions:**

Establishment and implementation of national suicide prevention strategies will not be possible without reliable statistics and comprehensive standardized reporting practices. The case of Nepal points to the need for collaborative reporting and accountability shared between law enforcement, administrative, and health sectors. Awareness of legal codes among health workers, in particular dispelling myths of suicide’s illegality, is crucial to improve mental health services and reporting practices.

## Background

Suicide is one of the fastest-growing and least-understood causes of death, particularly in low and middle-income countries [LMIC] [1, 2]. Globally, suicide accounts for one million deaths per year, with the majority of the burden in LMIC [2]. The World Health Organization (WHO) Action Plan calls for a reduction of suicide mortality by 10 % globally [3]. This initiative and other efforts to reduce mortality and morbidity associated with suicidal behavior require quantitative evidence to reliabily document the severity of the problem, the major risk groups involved, and the impact of prevention efforts. This practice—public health surveillance—has been defined as “the systematic, ongoing collection, management, analysis, and interpretation of data followed by the dissemination of these data to public health programs to stimulate public health action,” (page 3) [4]. Suicide and suicidal behavior, however, are notably challenging to reliably track in surveillance systems. The difficulty in documenting suicide has contributed to the lack of initiation and sustainment of suicide prevention programs globally [5]. Worldwide, violent death reporting systems require significant improvement to provide reliable data to design and implement programs [6, 7]. LMIC in particular lack the capacity to accurately track such deaths [8]. High-income countries also struggle to accurately and timely capture violent deaths [9–13]. Violent death surveillance systems are often complex, requiring the coordination of health, legal, and administrative systems, as well as the cooperation of families and informants [14, 15]. Mental health systems are emerging in LMIC, and this is an ideal time to examine suicide surveillance and its incorporation into mental health system strengthening and other development strategies [16–18].

State-generated official estimates of suicide are largely under-representative due to misclassification, stigma, and little technical capacity [19–22]. Suicide data are filtered through reporting systems shaped by social, cultural, legal, and medical institutions. Bureaucratic documentation serves many roles including categorical derivations of disease and cause of death [23–27], exertion of global economic governance [28], power distribution [29–31], and validation of personhood [32, 33]. Macro-level (e.g., multi-national agencies, often deployed as development organizations such as USAID, UNICEF, etc.) and institutional labeling practices (e.g., cause of death, disease classification, birth/death counting) frame experiences at lower social levels, including the community, family, and individual [8, 26, 34].

Nepal represents an important example for the challenges of suicide surveillance. Recently, the suicide rate among women 15–45 years old in Nepal was found to be 28/100,000, and suicide is the leading cause of death in this demographic group, accounting for 16 % of mortality [35]. Compared to the female suicide rate in the US (5.5/100,000), the speculated burden is incredibly high. Precipitants were postulated to include lack of education, being married, poverty, gender-based violence, and belief in *karma*; however, these findings are limited to a small sample of women so that little is known about male suicides [36].

Despite growing recognition of the high burden of suicide deaths in LMIC [2] and an alarming level of suicide among Nepali women specifically [35], Nepal’s Ministry of Health does not systematically collect nor report on suicides [36–38]. In 2011, Pradhan [36] described the incongruent reporting between health and law enforcement data systems within Nepal’s institutions responsible for capturing information related to death. Given that women’s suicides in Nepal are perceived as a public health crisis, the lack of systematic documentation processes to characterize the prevalence and risk factors is striking and of urgent concern to public health and international agencies. According Pradhan and colleagues [36], suicide is illegal, and the legal system is responsible for capturing, charging, and reporting all such deaths. Nepali police report some suicide deaths, however a follow up study found large institutional differences in reporting practices. For instance, in 2010, police records officially report far more suicide deaths (3990 suicide deaths) than the Ministry of Health (6 reported deaths) [39]. Additionally, they reported many more male suicides than female, which contrasts with community based findings of high rates among women. As there is not yet a comprehensive vital registration system in Nepal, national level suicide data are not systematically collected and no suicide mortality data are reported by the World Health Organization [40, 41]. However, in 2014, the WHO has modeled a 2012 predicted suicide rate for Nepal, ranking it 7th in the world at 24.9 per 100,000, the 3rd highest for women (20 per 100,000), and 17th for men (30.1 per 100,000) [42].

To date, despite one report of a remarkably high suicide burden in Nepal, no such studies have investigated state-generated data nor the role central level governance systems play in the detection and reporting of suicide deaths and attempts. Suicide deaths are unique in that they are documented both as a legal and health issue. Communication across agencies is essential to better address a poorly understood leading cause of death worldwide. This study extends social network methods through a qualitative lens in an effort to provide critical insights into the system level actors charged with detecting and reporting such deaths. Such an approach can uncover ways in which institutions impinge on prioritization and perceptions of suicide as a health problem through dominance, discrimination, power, and control [43].

The current study seeks to better understand barriers to suicide reporting in Nepal and challenges in the data chain linking self-inflicted deaths to Ministry of Health and Population documentation and ultimately to international reporting for global institutions such as the World Health Organization. Following the model of similar studies which have incorporated anthropological methods to investigate health system documentation and reporting [44–48], we conducted in-depth interviews with stakeholders involved in various levels of the death reporting systems in Nepal to explore the perceptions, practices, and politicization of suicide reporting. Pictorial depictions of surveillance pathways were also elicited to gain a more textured understanding of how reporting frameworks were believed to function across varying institutions (legal, health, development, and community). This study seeks to analyze networks drawn by police, policy, and health officials in order to better understand vital surveillance in Nepal and investigate how institutional networks affect how suicide deaths might be (un)documented and (un)reported within varying institutions. This study offers a novel approach to understanding health and information systems in a resource poor setting, where infrastructure and documentation are limited.

## Methods

### Setting

Nepal is a low-income, post-conflict, South Asian country with a population of 27 million people; it is also a country disproportionately impacted by suicide compared to high-income countries and LMIC in other world regions [1]. 80 percent of Nepal’s population resides in rural areas often is characterized by poor access to physical health care, limited mental health services, and high rates of poverty and illiteracy [49]. Maternal and child health outcomes are poor (Suvedi et al. [35]), and women often lack agency in Nepal’s patriarchal society and are often victims of domestic violence [50]. In addition to chronic socioeconomic and health problems, the country suffered a decade-long civil war from 1996 to 2006, halting health system development and further straining relationships between communities and police officers. The country’s capital, Kathmandu, was chosen as the study site as it houses the central-level government facilities and international organizations responsible for health and legal system development and management. Nepal’s health system is still developing and remains dependent on foreign development aid and private investment [51].

### Sampling and methodological framework

Ethnographic fieldwork was conducted in Kathmandu, Nepal. Fieldwork consisted of participant observation in health, development, and legal institutions as well as key informant interviews [52]. Health system and legal professionals were recruited through purposive sampling in order to elicit popular constructions of how the current death surveillance system functions, where perceived gaps may exist and potential strategies to improve the coverage, accuracy, and functionality of the system. The sampling procedure sought to maximize variation across institutions involved in fatal events (police force, government health system, Ministry of Health and Population, clinical caregivers) and institutional levels. The sample was sufficient to stratify by institution [53]. Levels were stratified into three categories according to Singer and Baer’s critical medical anthropology and world systems framework: (1) the macro (multilateral agencies that enact power across global systems), (2) the meso (large national bodies that oversee country priorities and polices and may communicate with macro bodies), and (3) the micro (organizations operating between country and community levels) [54, 55]. For the purpose of this analysis, macro-level agencies were labeled as ‘development agencies’, per the common development and assistance agenda such institutions employ.

### Semi-structured interviews

Thirty-six semi-structured interviews were conducted across law and health system levels including multilateral organizations (WHO, World Bank, Institute of Migration), foreign aid agencies (DfID; USAID), government ministries (Ministry of Health and Population, Ministry of Home Affairs), healthcare institutions (government hospitals, academic hospitals), legal and law enforcement institutions (district police, national police academy), and nongovernmental organizations (mental health and psychosocial organizations, advocacy organizations) (Table 1). Interviews elicited perceptions of the existing surveillance system, communication across and within institutions, how suicide cases are handled and documented, existing challenges hindering optimal system functioning, and specific suggestions for system improvement. State-generated data (including the Nepal National Police and Ministry of Health annual reports) were also reviewed to assess morbidity and mortality indicators related to suicide.

**Table 1:** Semi-structured interview participant characteristics

Subject#	Category	Facility	Occupation	Gender	Age
1	Administrative	Government Ministry	Statistician	Male	40–49
2	Administrative	Government Ministry	Surveillance director	Male	40–49
3	Administrative	Multi-lateral organization	Program researcher	Female	30–39
4	Administrative	Multi-lateral organization	Department director	Female	40–49
5	Administrative	Multi-lateral organization	Program coordinator	Female	>50
6	Administrative	Multi-lateral organization	Development officer	Male	30–39
7	Administrative	Multi-lateral organization	Technical advisor	Male	30–39
8	Administrative	Multi-lateral organization	Technical advisor	Male	30–39
9	Administrative	NGO	Researcher	Male	30–39
10	Administrative	NGO	Researcher	Male	30–39
11	Administrative	Village Development Office	Data clerk	Female	40–49
12	Health	Academic hospital	Psychiatrist	Male	40–49
13	Health	District Health Office	District health officer	Male	>50
14	Health	Government Ministry	Statistician	Male	40–49
15	Health	Government Ministry	Director	Male	40–49
16	Health	Government Ministry	Information management	Male	40–49
17	Health	Government Ministry	Epidemiologist	Male	>50
18	Health	Government Ministry	Retired official	Male	>50
19	Health	Multilateral organization	Psychosocial lead	Male	30–39
20	Health	National Hospital	Head nurse	Female	>50
21	Health	National Hospital	Psychologist/professor	Female	40–49
22	Health	National Hospital	Psychiatrist resident	Male	30–39
23	Health	National Hospital	Psychiatrist	Male	40–49
24	Health	National Hospital	Professor and psychiatrist	Male	>50
25	Health	National Hospital	Psychiatric resident	Male	30–39
26	Health	NGO	Psychologist	Female	30–39
27	Health	NGO	Program manager	Female	30–39
28	Health	NGO	Psychological counselor	Female	30–39
29	Health	NGO	Psychosocial worker	Male	40–49
30	Health	NGO	Researcher	Male	40–49
31	Health	Private Hospital	Director	Male	40–49
32	Legal	Lawyer	Lawyer	Male	20–29
33	Legal	Police	Division director	Female	30–39
34	Legal	Police	High-ranking official	Female	40–49
35	Legal	Police	High-ranking official	Male	40–49
36	Legal	Police	Police officer	Male	40–49

Interviews lasted between 1 and 2 h and were conducted in either Nepali or English at the preference of the respondent. The first author conducted all interviews and was accompanied by a Nepali research assistant fluent in English. The research assistant was trained in interview and translation protocols as well as ethics. All interviews were transcribed into English, systematically coded, and analyzed in MaxQDA 11. A codebook was developed to identify common themes and coded all textual data as outlined by Bernard [56]. Thematic analysis was employed to identify typical and atypical examples of each theme [52]. Data validation was conducted by having team members scrutinize themes, their descriptions, and exemplars. Nepali colleagues were consulted to verify our interpretation of the data. Text analysis of the accompanying interviews provided context, descriptions, and elaborations surrounding the current system in Nepal and perspectives on challenges and improvements across sectors.

### Descriptive social network analysis

Social network analysis can be used to not only show the ties among actors in a system, but also the impact that relationships among actors can have on decision-making, flow of information and the overall structure of a society [57–60]. Only recently has social network analysis been employed to better understand the supply, demand, flows, and social dynamics of information within health systems [61]. Particularly in developing contexts, such as Nepal, it is important to understand how increasingly complex information is being collected, assessed and shared. Social network analyses within health systems can offer unique insight into where information might be shared, lost, or biased.

For network analysis, two research questions were explored: (1) What institutions ‘create’ and ‘control’ data transfer and communication of suicide deaths? and (2) how do perceived death surveillance networks vary across institutional informants?

To gain a better understanding of the formal and informal health surveillance system, a sub-sample of key informants (n = 23) were asked to hand draw, to the best of their knowledge, how deaths due to suicide were differentiated and documented. They were asked to include data pathways and repositories so that comparisons could be made between the ‘official’ death surveillance system with how it is understood and followed by those implementing it. Finally, subjects were asked to note the communication pathways suicide data traveled through, from initial informant interviews (discussion with family/surrounding community) to the ‘final’ resting point of data. This process provided insights into perceptions of formal data collection processes across institutions, as well as perceived opportunities and challenges amongst communication and data sharing pathways.

A codebook was created to indicate the institution (police, health, or community) and the level at which the institution worked (macro, meso, micro). Informant-generated pictorial data were coded for actors included in the informant’s network and noted for which institutions were responsible for nodes of data collection and transfer. All codes were translated into a numeric ID that was consistent across all networks. An edge list was then created to indicate each directed ‘tie’ within the informant’s perceived network. The resulting edge list was uploaded into R Studio Version 0.98.1062 as an array of 23 matrixes, each representing the digitized version of the hand-drawn network. Each matrix contained 13 by 13 possible institutional ties. Multiple ties were possible. Networks were descriptively assessed for presence/absence of institutions and centrality of actors as conceptualized and measured by Freeman [58, 62]. To determine the extent to which each institution was involved in the ‘control’ of suicide-related data, betweenness was calculated for each institutional category within each network and then averaged across informants. In social network analysis, betweenness is a commonly used measure that indicates how much a node is located on the path between other actors [58]. Nodes serving as a ‘bridge’ or ‘connector’ between many other actors are considered to ‘control’ information and communication within the network.

### Ethics approval and consent to participate

All aspects of the study received approval by Arizona State University’s Institutional Review Board (STUDY00000945) and the Nepal Health Research Council (NHRC Reg. No 290/2014), the government body responsible for authorization of health research in Nepal. Participants provided written consent for participation in the study and the publication of the findings. The research was conducted in collaboration with Transcultural Psychosocial Organization (TPO) Nepal, a research-oriented nongovernmental organization headquartered in Kathmandu.

## Results

### Overview of reporting and documentation process

**Fig. 1:**
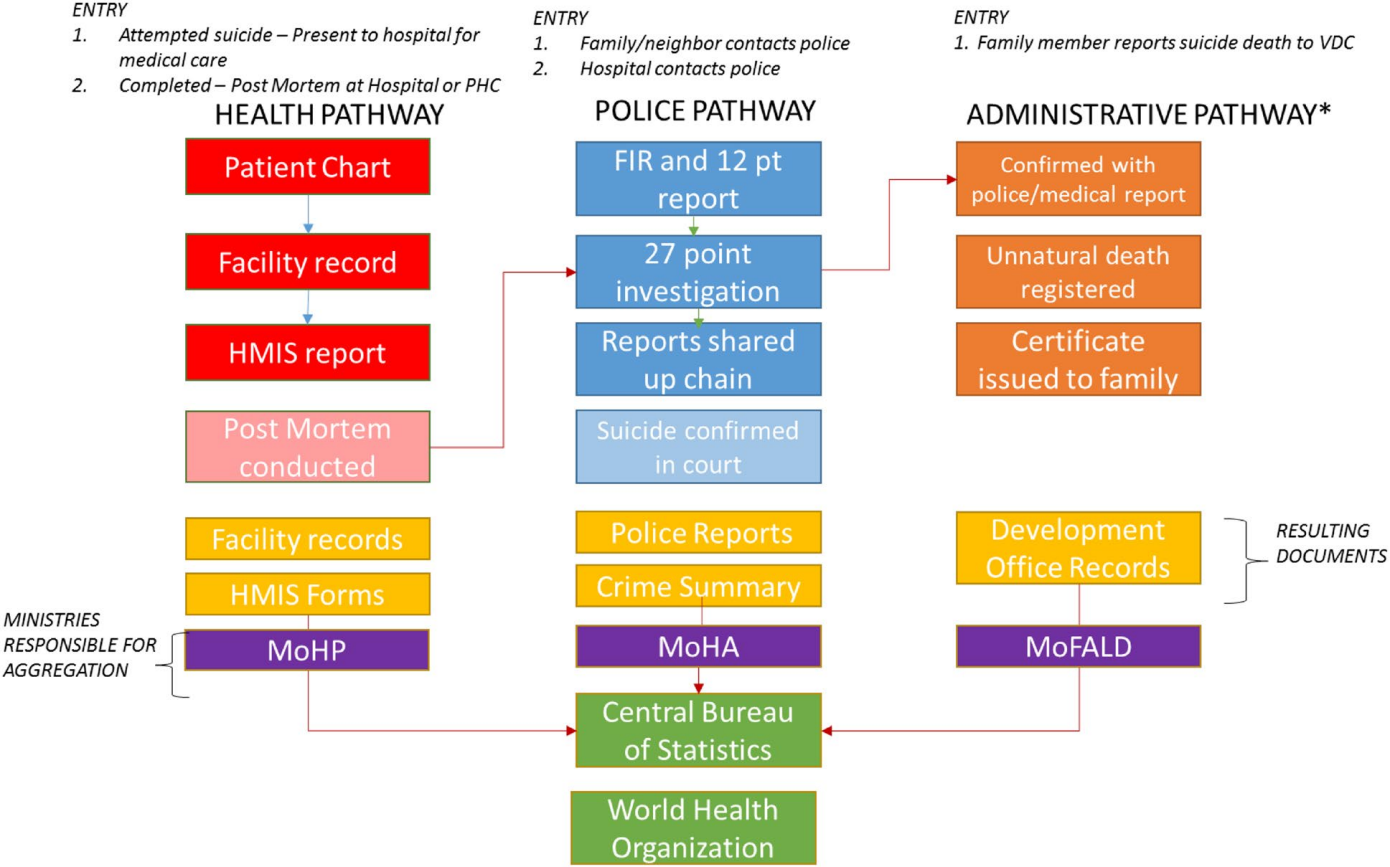
Reporting pathways through governmental health, police, and administrative systems

According to the Nepal Ministry of Health and Population, health data are aggregated from the local health post, to the district health office, and then sent monthly to the Health Management Information Section (HMIS) in Kathmandu where they are converted into electronic records. Paralleling this system, is an administrative pathway. All births and deaths are to be reported to the local ward office, village development committee (VDC) or municipality office, depending on proximity. To confirm the death, a staff member comes to the home of the deceased where the reporter must collect at least seven individuals that personally knew the decedent. The death is then certified in front of the seven witness and reported back to the VDC office. These reports are aggregated monthly and sent upwards to the District Administrative Office, and, eventually, the Central Bureau of Statistics. From here, many developing countries report all births, deaths, and other important health indicators to the World Health Organization; however, Nepal is not currently able to enact this reporting pathway because a formal vital surveillance system does not exist. The reporting pathways for a suicide death are outlined in Fig. 1.

**Fig. 2:**
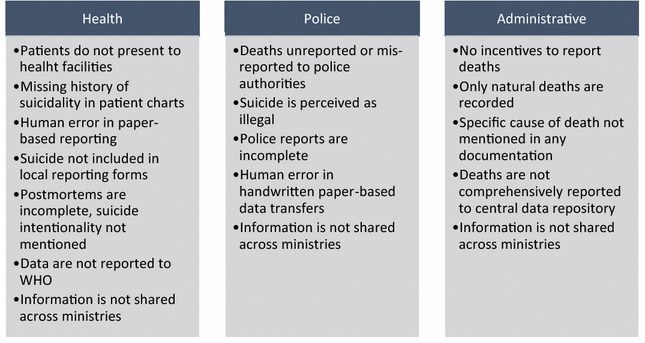
Summary of barriers to reporting pathways for suicide deaths

**Fig. 3:**
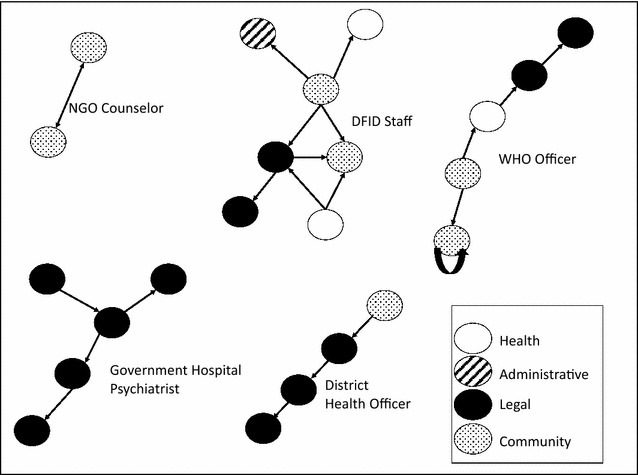
Digitized hand-drawn surveillance network samples from five informants

Based on the interviews with police, members of the health system, government workers, and other stakeholders, we extracted information on stakeholder roles, the legal requirements for that stakeholder group, the group’s definition for suicide (if any), and the barriers to documenting and reporting suicides (see Fig. 2; Table 2). Examples in the variation in perceptions of reporting practices are displayed in Fig. 3.

**Table 2:** Documentation procedures, policies, and practices as reported by stakeholders

Stakeholder group	Roles with group	Legal requirements related to suicide	Policy providing guidelines	Definition of suicide	Reported experience
Nepal Police (Ministry of Home Affairs)	Local police intake individual report and relay to the district police office District police investigate, document, request a post mortem, and send case files to the central headquarters. The court system confirms suicidal deaths after two years	Investigative reporting, documenting witnesses, submitting case file and evidence to the court system	Section 187: Draft Penal Code (abatement of suicide) Country Code (Muluki Ain): Procedural Investigation for homicides and unclear suicides No written laws exist criminalizing an individual for dying or attempting suicide in Nepal	No explicit definition in the Penal Code nor the Muluki Ain	Individuals do not report suicides to police Homicides are misreported as suicides Officers are bribed to not record death Paper-based documentation causes human error and data loss Reports are not properly completed Police occasionally have to pay for health and post-mortem related fees Officers are not trained in suicide first aid or how to best interact with the family during investigations
Health system (Ministry of Health and Population)	DEATHS: Conduct post-mortem procedure and immediate cause of death Appear in court as a medical expert ATTEMPTS: Nurses must maintain the records documenting the patient’s diagnosis according to the physician Physician: treat and stabilize attempt case Psychiatrist: See all ‘accident cases’; talk to family and patient, perscribe medication, determine severity of intent, give psychiatric diagnosis, and document changes in suicidal thoughts and behaviors FCHV: monitor births and deaths and submit monthly report ot the health post Health post: aggregate monthly reports from FCHV and health post services. Send data to district health office (DHO) DHO: aggregate data from health posts and send to the national level ministry	DEATHS: Conduct post mortem report and confirm death with an EEG ATTEMPTS: No legal requirements	Standardized reporting forms designed by MoHP Only government institutions can perform post mortems. Physicians must appear to court if called Inexperienced doctors are encouraged to not speculate on underlying cause of death	lCD codes (X60, X68, X70, X78) Post mortem report requires immediate cause of death but no underlying cause	Suicides do not present to the health sector Suicides are misreported as accidents or homicides as suicides Suicide attempts are included as an indicator in HMIS forms and thus never recorded Emergency room data are not included in MoHP reports nor the medical record office Dead on arrival cases are not reported in hospital data The quality of health post data is low and reports are often not submitted FCHVs do not actively report deaths
VDC or Municipality (Ministry of Federal Affairs and local Development)	Issue death certificates. If death is reported as suicide, the office requires a police investigation and doctor's report	All unnatural deaths must have supporting police and medical reports. Send aggregated data to the District Development Office	Civil Registration Act of 1976	Police and medical documentation related to the cause of death	Individuals never report a death to VDC as suicide Deaths are typically only registered if a certificate is needed for property transfer or other government services
Other government institutions	Central Bureau of Statistics is charged with collecting and reporting data from the police, MoHP, and other ministries Department of Home Affairs: aggregates police reports Ministry of Federal Affairs and local development: conducting and reporting births and deaths

## Institutional roles and procedures

### Role of police in confirming suicide events

**Fig. 4:**
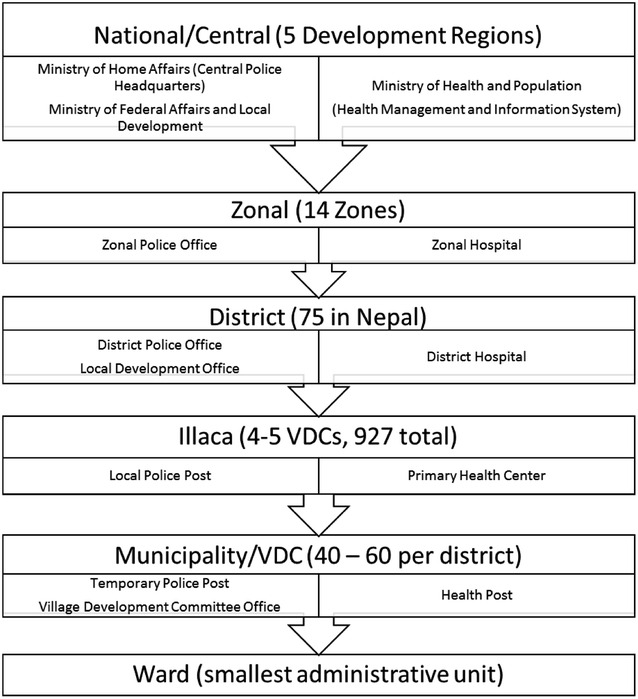
Governing units of Nepal with corresponding police, administrative, and health institutions

The Nepal Police, who are under governmental body of the Ministry of Home Affairs, are tasked with determining suicide death and reporting them [63]. Per the *Muluki Ain* (Nepal’s General Code outlining all civil and criminal law), there is no provision criminalizing, defining, nor punishing suicide. It does make reference to the procedural investigation of all homicides and suspicious suicides. Although suicide is not illegal, suicides were consistently reported as a ‘criminal’ and ‘legal issue’ by the majority of health informants, and the confirmation, investigation, and communication with the family and victims is mainly conducted through the police, with little input from clinicians. District and national police officers depicted the informational chain as originating with a family or community report of a death followed by the initiation of formal procedures to collect evidence (both physical and verbal). Police then complete several reports (an initial brief inspection report detailing the day, time, location, and individuals involved, a subsequent longer report, a request for a post-mortem report from a certified hospital, and a full report with complete case details that can only be completed after the case is closed. Reports are transcribed at the district office and sent via post or email to the Zonal Police Office, the National Police Headquarters in Kathmandu, and the District Court through the Attorney General (see Fig. 4 for governing units of Nepal). Districts may vary in documentation sharing processes, whereby some are hand-written and hand delivered and others must travel through the post. Local reports are often collected over phone/radio from a remote ward and then input by a clerk at the District Headquarters.

A police officer explained the procedure, “We put the investigation reports in our register. We have a very big register and we keep all the data in that book. It’s all on paper. That’s the major problem here. I was scared, when you asked me about suicide. I thought, ‘Oh my god, now I have to ask somebody to count everything.’” Although one district office stated that all the records were input into computers, they always handwrote a report, citing the mistrust of easily manipulated electronic records and unreliable and inadequate electricity that limits use of computers. Reports remain in the District Police office for 2 years in a folder, after which the case is officially closed and reports stored in sacks on top of filing cabinets. Every police officer interviewed stated that the police captured “all unnatural deaths,” even in remote rural areas. If a family did not want to report a suicide, the neighbors would notify the police because it was “the law.”

A suicide case and all subsequent reports require signatures (or finger prints if illiterate) of five witnesses, although police investigators stated that three is sometimes sufficient. These are usually the family and neighbors of the deceased. The process is exhausting and time consuming, for both the family and the officers, and often results in unanswered sections of police reports, particularly the history of the decedent’s death. A high-ranking police official noted that a big challenge with the documentation process was that local police officers fail to properly investigate suicides:

The local officers are not serious when collecting the data for a suicide case. There is no training at all on how to respond to suicide. Because we don’t have training, because in most of the suicide cases we don’t know the reason, the officers don’t write it. Our police in the field, many are not doing it [because], it is so easy to [instead] write that they ‘could not find the motivation for suicide.’ The form is very good, but they fill it out very badly. They don’t bother to know the reason behind the suicide. Even when I ask them, they say, ‘Oh, there is no suicide note, so how do I know why she did it?”

Some officers went on to discuss the lack of training in the force regarding how to deal with sensitive cases like suicide. There is “no proper training to handle the suicide case. That would be helpful. Our police officers should get counseling training, how to make the witnesses more comfortable. But it requires time and patience, and that is hard.” These issues result in incomplete case reports and a limited picture of suicides within particular districts, particularly in rural areas.

Finally, officers pointed to one of the biggest challenges with confirming a suicide or determining the intent, as many families tried to hide, mask, or blame others for a death. One high-ranking police official explained the complications with families, particularly in the case of female suicides.

There is always a dispute in case of suicide in women. If there is a girl, or domestic helper, or daughter-in-law, and she committed suicide, then there will be an issues. Like, maybe she was raped and murdered, maybe the in-laws killed her or hanged her. These issues are raised normally amongst the families involved, and it is very, very-very difficult to differentiate, and sometimes there will be very, very-very huge pressure so that sometimes we have to arrest in-laws. We don’t have choice.

The officer then explained the case of a domestic helper who died by suicide. Immediately, activists surrounded the police station and gave massive pressure, “so we had to arrest the house-owners for suspected murder, even though it was a suicide.” The pressure exerted by the public had clear influence on the way police cases were handled in urban areas.

### Medical-legal cases: the minimal role of the health sector

The health system’s role in suicide-related cases was reported to be three-fold: (1) *clinical care* for live cases following an attempt; (2) *clinical examination* of the dead body for post-mortem; and (3) *data collection and reporting* related to suicidal behaviors. The latter is the responsibility of Nepal’s Health Management and Information Section (HMIS) under the Ministry of Health and Population (see Table 3 for existing HMIS indicators related to suicide).

**Table 3:** Suicide-related and unidentified intent indicators in Nepal’s health management and information system

ICD-10 code^a^	Disease description
X60	Intentional self-poisoning by exposure to non-opioid analgesics, antipyretics and antirheumatics
X68	Intentional self-poisoning by and exposure to pesticides
X70	Intentional self-harm by hanging, strangulation and suffocation
X78	Intentional self-harm by sharp object
T60	Toxic effect of pesticides
T62	Toxic effect of other noxious substances eaten as food
T65	Toxic effect of other and unspecified substances

^a^Burn and other detailed injuries not included. All self harm indicators are included

We interviewed several top-ranking officials within HMIS to better understand how suicide information was, or was not, collected, prioritized, and distributed. Currently, HMIS does not systematically report suicide deaths. When asked if such an indicator was important, a high-ranking health official replied:

I have been here [HMIS] for 15 months, and no one has asked about suicide; no one has asked why there is no data, so I do not know. This is the first time I have heard about this, and I have worked in health for 3 years. I remember, I presented this [suicide] report in several forums in health planning meetings, but after the discussion, nobody was asking or willing to know; there was no interest or recommendation to capture suicide data in the system. Among 10 causes of death, we know it is number one for women, but the other nine are where everyone is concentrating on, like calcium deficiency. Maybe I will explore it, but no one is willing to do this research or finding the cause.

When prompted to describe the health system’s role in data collection related to suicide deaths, most informants initially touted that it was the police system’s responsibility to collect such data. The term ‘medical-legal’ was used constantly in our conversations to emphasize that, although this was a death, it was one that ‘belonged’ to the police, not the physician. Doctors specifically discussed how complicated it was to ‘record’ such deaths, and that they were legally not allowed to make such a call. Suicides often only appeared in the health system if they were brought in for a post-mortem, however, only a few health centers (Government District Hospitals and some Primary Health Centers) are qualified to conduct such exams. Oftentimes, young physicians staffing rural primary health centers are charged with conducting all the post-mortems for the police, and rarely are they specifically trained in how to identify suicidality. Moreover, clinicians insisted that it was not ‘legal’ for them to mark the ‘cause of death’ as suicide, as that was the responsibility of the police. Rather, they were simply charged with identifying the manner of death (e.g., cardiac failure, asphyxiation). In fact, informants that were tasked with post-mortems in their early career cited that the great majority of all deaths they assessed were suicides. Some clinicians that performed autopsies agreed that they could not speculate on the intention related to the death, as they only “saw the dead body in the exam room.” However, others mentioned that it was often easy to tell if the wounds were self-inflicted or not, but they had no power to make such a declaration. Despite physicians having the ability to discuss circumstances with family members for more information, this communication platform was rarely pursued.

Because the autopsy reports were ‘medico-legal’ documents, they ‘belonged to the police’ and were not included in the HMIS report. When asked if the informant believed these should be integrated into HMIS, all unanimously agreed ‘yes’ but then cited complications of implementing such a rule. Doctors, more than any other group of informants, declared that there was no reporting system for suicide cases. A psychiatrist laughed and stated, “In Nepal, there is no reporting system. Suicide cases are only reported if it goes to the police. Maybe that happens in the big hospitals, but it’s rare in the community.” He went on to describe why it is so difficult to integrate private institution data into HMIS:

In Kathmandu, urban hospitals are supposed to report the cause of death, but the majority of healthcare is not provided by the government hospital. So, the private hospitals are not advised to accurately report to the government. Most of the hospitals don’t have internal recording systems and don’t have way to report it either. Our clinic [is] supposed to report every 3 months, but there is no check and balance. Even now there is no proper medical record which includes the name, cause of illness, treatment, and advice given to the patient. We don’t keep medical records, so when it comes time to report, I have nothing to go off of. So I just report what I remember. An HMIS mechanism should exist, but it doesn’t, not in the private clinics.

While HMIS does have ICD codes within their system for suicide deaths, the HMIS does not systematically collect or report self-harm or suicide data within their system. This is despite their ability to do so because all suicides must legally have a post-mortem, and all post-mortem procedures have to take place in a government medical institution. However, clinicians and health officials we interviewed did not know ICD codes existed for such deaths, highlighting a gap in the ‘official’ system whereby the infrastructure may exist, but individuals filling out paperwork are unaware. While the purpose of the study was to document death surveillance, interviewees also emphasized the complete lack of documentation within the health information system relating to suicide *attempts*.

Most health informants reported that no suicide death recording system existed at all within the health system. One multilateral health organization official stated, “You see, HIV has a specific program, a funded surveillance system that is completely separate from most of our reporting. So all the HIV cases and deaths, they get reported. But suicide, mental health, there’s no division [departments], no funding for that, so none of those deaths are captured systematically.” Often, clinicians highlighted the haphazard documentation of any patient cases, citing that the data were never utilized by anyone anyway, so there was no value in detailed documentation. For instance, post-mortem reports are kept both in police records and in the hospital records, but not one clinician noted ever aggregated the information or used the death data to inform their own work. The post-mortem reports were often cited as being conducted “for the police,” indicating their value within the health system was negligible. One physician at a large government hospital noted, “The recording of [suicide] cases, it’s not so strictly regulated. I think in Nepal, we do a lot of things, but we do not record it or do any analysis of it […] Record keeping is the poorest thing that we do. Definitely”.

Moreover, doctors admitted that all cases are certainly not brought into the health system, so HMIS cannot possibly capture the true burden. Even bringing in dead bodies for a post-mortem is challenging, as the burden for transport and related costs are borne by the family. One physician commented that when he worked in a rural district, many cases were not ever brought in because it might take days and thousands of rupees to move the body. Families could neither afford the fees nor the time, as the bodies should be burned as fast as possible. At the community level, Female Community Health Volunteers (FCHVs) are tasked with tracking all births and deaths within their catchment areas. However, a leader at a large INGO stated that FCHVs miss such events all the time, as they are preoccupied with maternal health related issues. FCHVs are incentivized to report and bring births into the health system, however, death reporting is not compensated. Moreover, the FCHVs are often focused more on women and children than men, so their documentation would likely miss any male suicide events entirely.

A former official within the Ministry of Health and Population explained that the health system’s relative neglect of mental health to date may stem from the practice of the Joint Annual Review (JAR). This high-level meeting consists of macro level organizations (including WHO, World Bank, DfID, USAID, etc.) and Nepal’s Ministry of Health discussing the progress and future priorities of Nepal’s health sector performance. When prompted to discuss whether mental health is discussed at these meetings, a retired government health official replied, “(*laughing*) Always. It’s the same story. It [mental health] should be integrated. But the health ministers, they should be serious about it, no? It is always discussed but never acted on. The ministers come and they only look for their own area, ‘What can I do in my area?’” Political influences were believed to heavily affect the ministry’s priorities, leaving localities with historically little funding from development agencies, undeveloped indicators, and no immediate perceived need in ministers’ districts without a spot on the upcoming health agenda. Finally, despite interviewing several government and international development informants, no one could describe how data get to the WHO or why suicides might not get reported, besides the fact that HMIS was not responsible for reporting such deaths.

Despite suicide recording, and health information recording in general, not being a high priority for the health respondents, some units did informally document suicide cases. In one patient record book, nurses indicated with a red ‘S’ if a case was suspected to be a suicide. The nurse explained, “No. We never mark a case as suicide. Not on the death certificate or in our reports. That’s the police’s job. We do sometimes indicate in our patient log a red ‘S’ if we suspect a suicide. But this information stays with us. We do not send it anywhere.”

One of the most notable findings was the persistent misconception among health workers that suicide was illegal and a punishable offense. It was often commented that cases were not reported because of the legal consequences. One psychiatrist commented, “In Nepal suicide is a criminal offense, so lots of the cases they don’t get reported because of the stigma in the family and community, you know? They won’t want to get the police involved.” Additionally, health staff and other administrative personnel were hesitant to document many details because of the legal sensitivity. A nurse explained that, “we cannot write down suicide in any of the documents. Only the police can do that. We can write poison case, if that is what the doctor wrote, but we never mention the intent.” Another physician that conducted post-mortems mentioned that, “Many of the junior doctors, we are nervous to write suicide. It is illegal so we might be called to court, what if we are wrong? We let the police do that.” Lack of documentation and reporting was often attributed to this belief. This was in stark contrast to the actual legal code, the *Muluki Ain*, which contains no reference to suicide as a punishable offense.

### Role of the community and administration sector

Informants noted wide variation in the completeness, accuracy, and capability of the ‘surveillance system’ in Nepal, highlighting many needs and challenges for documenting a complicated cause of death. Deaths are recorded at the community level through Village Development Committee offices (VDCs) or Municipality Offices. VDCs issue birth and death certificates. When interviewing VDC Secretaries (government appointed positions), both of whom had been in their position for well over a decade, the informants had never issued a cause of death as suicide. One VDC secretary explained why this was the case: “The family reports the cause of death directly to us. If it is anything other than a ‘natural’ death, we must inform the police and they will need to issue the death forms. That takes time…it’s a big hassle. So if the family just says it was a natural death, even if the individual was in their 20’s, the process is very fast and easy.” Often death certificates are not sought after; in fact, most often only in cases where property and wealth transfer legally require a death certificate of the previous owner are such forms filled out. In Nepal, property is generally held by the males in the family, so female deaths are nearly never reported to the VDC.

The VDCs are charged with reporting up to the Ministry of Federal Affairs and Local Development (MoFALD), a government division that is independent from the health and legal sectors of the country. A high-level multilateral organization official emphasized that there are no platforms for information sharing among VDCs, local health posts, and local police offices, so information becomes fragmented and perhaps duplicated at the higher ministry levels. Multilateral organization officials also pointed to the varying priorities of the ministries and donor agencies as one reason death surveillance has not yet been prioritized: “Right now we are focused on documenting all births for maternal health indicators and vaccine coverage, things like that. There is funding there for that. So those are the priorities.” Often, high-level agencies were cited as controlling what information was prioritized and therefore recorded well. Without funding and checks and balances for deaths, and considering the complicated coordination needed among several different ministries, the pragmatics of accurately capturing something like suicides seemed impossible.

While police informants were confident that all deaths were investigated by their personnel without exception, community members interviewed insisted that suicide deaths would rarely be reported to the police for the following reasons: (1) it increased the time before death rituals could be initiated, (2) if reported to the police, the family might be suspected of murder, and (3) extra cost placed on family. One psychosocial worker explained, “These types of deaths (suicides), they do not get reported to the police. They are brought to the community, and they decide if it is suspicious of murder. Only in that case would the police be called. Otherwise, it is handled internally. The police, they complicate matters. They delay the funeral. It stays in the community”.

A Nepali multilateral-organization development worker discussed common mistrust of the police, stating that she would not believe what they report as they might be easily bribed: “Suicide is also, especially when it comes to domestic workers, women, suicide is such an excuse. It is clearly a murder or an abusive situation […] and the police do not always properly investigate […] I mean, there are cases that I do not trust the Nepal police, I don’t trust that all these cases are suicides.” A high-ranking police official mentioned that she knew of a doctor being bribed: “There was a case where they bribed 80,000 USD. The post-mortem report said suicide, but the dead body touched the ground, so the father-in-law, wife, and son were blamed for the woman’s death. Three people were blamed for murder by the community. It was a big issue. But at the end it was the post-mortem report that was official, where they bribed the doctor to say suicide.”

### Social network findings

To explore perceived reporting frameworks and subsequent variation across informants, each pictorial network was coded and digitized. Examples of informant networks can be found in Fig. 4. Nodes were coded to indicate institutional type (see Fig. 4). Most networks were linear, where information exchange passed in one direction, with few instances of reciprocal exchange between institutions. Networks ranged from two to seven institutions involved in information exchange related to suicide deaths. Large variation exists among the perceived networks. Some were simple and involved few actors, while others were very complex identifying many actors communicating across many institutions (Fig. 4). For example, a network drawn by a local psychosocial worker contained only two nodes, both at the community level, whereas the mean was over five, with some networks containing 10 nodes.

Informants that worked at a macro level (WHO, DfID, etc.) had generally larger and more complex networks compared to those working at the community level. For example, a psychiatrist working at a large government hospital drew a network where only police were involved in the data collection and suicide declaration process (Fig. 4). Despite denoting what the system was supposed to look like, many informants mentioned that the system was dysfunctional and disconnected. One multilateral organization official mentioned, “Everything is problematic here in the system. Our systems are not strong, forms are not completed per the WHO or UN recommendations, and the suicide deaths in particular are not well captured in the system. The police are the only officials mandated to document and declare suicides.”

To determine which institutions ‘controlled’ the data most (either through data generation, classification, and data ‘ownership’) indegree was assessed by counting the number of times a respondent indicated that a particular node received information from another node. Table 4 summarizes indegree by institution. Police had the majority of incoming data, suggesting that they ‘own’ and transform most of the suicide information into ‘fact’. However, whereas international macro-level institutions are ideally the final recipients of data, only four incoming ties were reported. Furthermore, the macro-level institutions identified for such ties were the Central Bureau of Statistics—a Nepali based institution that only receives data from the national police headquarters. No informants reported in their pathways that information would travel up to the World Health Organization, the global institution ultimately responsible for identifying health priorities and rallying for resources.

**Table 4:** Extent of ‘control’ across institutional category

	Police	Health	Community	Development	
Indegree, N (%)	46 (40)	36 (31)	30 (26)	4 (4)	
Outdegree, N (%)	37 (32)	33 (28)	45 (39)	0	

	Police	Health	Community	Individual	Macro
Betweenness, range (average)	0–4 (1.11)	0–3 (0.63)	0–3 (0.41)	0	0
Terminal position, N	18	4	1	0	1

Health bodies were also involved in much of the incoming information, but as indicated in concurrent interviews, although a body may come into a hospital, doctors are not allowed to declare suicides, nor is ‘suicide’ ever indicated on a death certificate. Only police have the ‘legal power’ to declare such deaths. One nurse even noted that many health professionals were nervous and scared to be involved in declaring suicidal cases—even if there was robust physical evidence to support such a cause of death—because it was not their ‘professional position’. The community, logically, has the majority of outgoing ties, suggesting that the source of data remains at the community level, which is ultimately responsible for initiating information sharing and official data collection.

Incoming and outgoing ties to each institution indicates how ‘central’ a particular node is to the network (i.e., controlling information transfer). These results are depicted in Table 4. Police had the highest average betweenness measures compared to other institutions. This indicates that the police are capable of ‘controlling’ the most information, as it has to pass through them more than any other node type. Individuals were extracted from their ‘community’ category to demonstrate that once individuals provide the original ‘data’, information does not route through them again and therefore, they have the lowest ‘control’.

Finally, in international ‘gold standard’ surveillance networks (World Health Organization, 1999), macro level institutions like the WHO are considered the ‘terminal’ point of data deposits (e.g., countries report their national data to the WHO, which then assesses global health trends). However, no informants indicated that information arrived at the ‘macro’ WHO headquarters within Nepal. After being probed, no informants could describe the mechanism by which WHO received country-based suicide data, why the WHO did not use the police data related to suicide, or what was required of the health system for WHO to begin reporting Nepal’s suicide rates. The highest level ‘terminal’ position informants mentioned was the Central Bureau of Statistics, an agency that collects data from the police headquarters and other government ministries.

## Discussion

Reliable and accurate suicide surveillance is crucial to design, implement, and evaluate suicide prevention strategies, with the aim of meeting targets such as WHO’s 10 % reduction in national suicide rates [3]. This study sought to better understand how suicide knowledge and information, specifically suicidal data, pass between and among institutions in Nepal. The key findings were:

1. A discrepancy between perceived criminality of suicide and actual legal codes;
2. The dominant role of police in collecting information, reporting suicide, and interacting with families affected by suicide;
3. A lack of systematic nationally standardized approaches within the health system for documentation and reporting of suicide, including limited communication channels between HMIS and global reporting (e.g., through WHO) of suicide statistics; and
4. Limited engagement of families in reporting suicide because of fear of legal entanglements anticipated with reporting suicide, anticipated stigma for families of suicide victims, and greater time and financial burden compared to reporting natural deaths.

Overall, there was large variation across the participant perceived networks, whereby some networks were linear pathways dominated by a single institution (police or community) with few nodes involved in data transmission, while others were complex and communicative. Such disagreement suggests disconnection amongst institutions. Yazdizadeh et al. [64] found similar disconnectedness and disagreement in knowledge networks in Iran. In Nepal in particular, the health system is known to be fragmented and dominated by vertical programs [65–67]. Despite recent recognition that improved surveillance systems in developing nations are crucial to improving human health [8, 68], little progress has been made [69]. Even in the United States, the National Violent Death Reporting System is only implemented in 18 states, indicating the difficulty of achieving robust information sharing processes [14]. Some lower income countries like India have official death registration systems, but they still face many challenges due to inefficient civil registration systems, variable standards for death certification, and under-reporting of deaths [70–72].

The findings show that some informants perceive the suicide reporting pathways to bypass formal institutions altogether. In these cases, it may be that community based surveillance systems will perform more accurately than relying on currently fractured systems. Cwik and colleagues successfully implemented such a system alongside the White Mountain Apache tribe using locally appointed counselors to visit families, conduct an extended verbal-autopsy, and report to the public health agency [73, 74].

In Nepal, one attempt has been made to pilot a robust health demographic surveillance system, but several challenges were encountered in order to maintain its sustainability including garnering political support and approval, geographic challenges, lack of household addresses, and very low death registration [75]. These challenges are reflected in this current study, where few deaths are officially reported. Additionally, cause of death is reported by the family with no requirement for a medical certificate. Families may be unaware or unwilling to report accurate causes due to lack of medical services, education, difficult administrative processes, and stigma.

Because only certain statistics (births, HIV/TB prevalence) in Nepal are reported to multi-lateral institutional bodies that hold the majority of health development funding, suicide and non-disease-specific deaths disappear from the global picture. This may further perpetuate the focus on current diseases that receive the bulk of international aid dollars (HIV, TB), as they are the most closely tracked and reported of all global health indicators. A recent study found that, although development assistance from international agencies has remained high, it did not align with recipient disease burdens [76]. HIV and maternal and child health remain the two most funded public health problems; however, mental health and health infrastructure lag far behind, particularly as mental health will soon be the leading cause of Disability Adjust Life Years (DALYs) worldwide [77–79]. Nepal’s health programs have historically targeted women and children, shaping funding patterns and program development to prefer gendered, female-based initiatives [80–82]. Better integrating suicide and other violent deaths into surveillance frameworks that are currently well established and funded within HIV and TB pathways may be one mechanism for increasing funding and success. However, the lack of alignment between disease burden, income, and funding reveals the need for improvement in resource allocation.

Importantly, social network analysis provides an important tool for exploring poorly understood and growing health systems, particularly in developing countries where documentation and monitoring is fragmented and infrequent [61]. Anthropologists have much to add to this discussion. Hull [83] argues that anthropologists have overlooked the role of bureaucratic documents, pointing to their mediative role in relationships and experiences of justice. Health statistics, particularly mortality data, produce ‘category fallacies,’ which result in the systematic misclassification of diseases and causes of death [26, 27, 45, 46, 84, 85]. When anthropologists situate official state generated data within the larger political milieu, prevalent health conditions may be overshadowed by priorities to prove ‘success’ or ‘need’ for the state to maintain funding [44, 46, 86]. Health system development in particular has been couched as imperialistic and neglectful of social and structural drivers [87, 88]. These studies, therefore, use a critical interpretive approach to understanding of death in ways ‘traditional’ numbers cannot [27, 89].

By exploring state-generated suicide statistics in a rapidly developing context (Nepal), the study expands upon, Erikson’s assertion that the production, use, and travel of health statistics are driven by geo-political-financial influences that shape perceptions, experiences, categories, and priorities of health [44, 90–94]. The current study demonstrates the complexity of death registration, and how particularly sensitive causes of death, like suicide, might be neglected from the health system and subsequent program planning all together. Nepal and other low income, high burden, countries may benefit from sparking collaboration between health, legal and administrative institutions at the community level. Increased communication about causes of death and subsequent program planning may initiate more confidence in documentation. Finally, demonstrating to local data recorders the utility of the data they create may improve the current documentation practices. Programs that can successfully use data to shape and improve outcomes may also inspire better and more connected data documentation and information sharing at the source of data creation, the community.

One final issue was the gendering of investigation, documentation, and reporting practices. Because of the popularization of the Maternal Morbidity and Mortality Survey that identified suicide as the leading single cause of death among women of reproductive age, as well as subsequent qualitative reviews emphasizing this issue [36], most health workers, policy makers, and other stakeholders reported attending primarily to suicide consideration when investigating women’s deaths. Agencies prioritized identifying and reporting female deaths, but often neglected to include men in such health program-planning. Ultimately, this framed female suicides as a health problem, but not male suicide mortality. This demonstrates how public health reports and subsequent media attention can drive incomplete and inadequate investigation and reporting practices. Prioritizing the development of a representative death registration system can help to provide timely and accurate information on causes of death so that development agencies do not rely only on verticle monitoring programs for specific diseases and populations.

### Limitations

The current study reveals important disagreements and misunderstandings of how the vital surveillance system *should* and *does* work in Nepal and offers important steps forward in order to begin to address systemic issues in the current operation of information sharing, both within Nepal and in the context of global development. Improved health information systems will further enhance the success of subsequent health improvement programs.

While the current study is novel and explores an under-studied topic, some limitations do exist. Informants often included both what they thought the system *should* be and how the system functioned in reality. This created inherent variation and ambiguity that is difficult to account for. Additionally, fewer police officials were engaged in the current research compared to health professionals. Similarly, the results presented in this article are only those from Kathmandu. Results should be interpreted with caution due to the limited variation of the study's sample. Finally, the analysis of the current project is highly descriptive and qualitative, and, as is typical of qualitative research, findings aimed at generalizability would need to follow a quantitative paradigm.

### Recommendations

WHO’s guidance on *Preventing Suicide* calls for formation of national strategies [2] in order to meet the targets for suicide risk reduction in the WHO Action Plan [3]. Findings from the current study suggest the following recommendations for Nepal and other LMIC settings:

1. *Raise awareness among health workers and international public health researchers and policy makers about medico*-*legal issues regarding suicide.* Health workers, law enforcement, and international development organizations should be versed in national legal codes related to suicide, as legal codes will affect reporting and documentation. A recent systematic review of 192 countries found that 25 countries have legal provisions making suicide illegal, and an additional 20 countries follow Islamic/Sharia law in which suicide attempts result in jail sentences [95]. Nepal was not among the countries in which suicide attempts are criminalized. We similarly found that that suicidal behavior is not illegal in Nepal, a conclusion reached based on a review of the *Muluki Ain* legal code and interviews with multiple lawyers and police officers. This finding conflicts with many publications and reports written on suicide in Nepal stating that it is a punishable crime [35, 36, 39]. For example, one recent review of suicide in South Asia claimed that suicide was illegal in all South Asian countries except Sri Lanka [39]. However, legal review of South Asian policies revealed that suicide is illegal in Sri Lanka, Bangladesh, Pakistan, and, until recently, India [95]. Misinformation in peer-reviewed literature is problematic in that it propagates false information and perpetuates stigma-inducing rumors. Misinformation about suicide’s legal nature by public health researchers reinforces barriers to appropriate reporting and mental health services. Through the education of government officials, particularly police, forensic doctors, and other officials involved in suicide death documentation and communication with families, myths and subsequent stigma may be dispelled. Additionally, the education of media professionals about the legal status of suicidal behavior can also enhance community awareness of both suicide prevention resources and non-stigmatizing information related to suicides.
2. *Collaborative, multi*-*sectoral approaches, especially partnerships between law enforcement and the health system are needed for reliable and accurate surveillance, and ultimately for effective suicide prevention.* The WHO *Preventing Suicide* report calls for multi-sectoral partnerships and the findings from Nepal illustrate the shortcomings when such partnerships are not in place. The lack of coordination and communication between law enforcement and health systems has led to potentially inaccurate estimations of suicide prevalence and has impeded collaborative prevention efforts. Lack of partnership between these groups also likely contributes to multilateral organizations, such as the WHO, not receiving representative statistics across reporting stakeholders. Collaborative teams that involve law enforcement, legal representation, mental and physical health, and other social services is now the rule for appropriate responses to gender-based violence (GBV). For example, persons affected by GBV can receive services in “one-stop” centers that integrate all of these sectors. Similar approaches could facilitate improved reporting of suicidal behaviors, as well as help assure that affected persons can receive care to prevent future behaviors and risks in family members. Collaboration can also support shared accountability, rather than approaches to shift to only health care involvement. By keeping law enforcement engaged this can address issues, such as the suspected high co-occurrence of suicidal behavior among victims of GBV. There is increasing precedent for effective law enforcement-mental health collaborations in LMIC, such as the Crisis Intervention Team (CIT) model which has been successful in Liberia [96], which is also a setting in which police have been the default party involved for attempted suicides.
3. *Suicide registries should be established which allow direct entry of information from various health, law enforcement, and social service sectors.* Exclusive ownership and accountability for reporting that falls only into the purview of one sector may underestimate certain types of self-injury and certain risk groups. A recent systematic review of suicide data and policies in South Asia points out the lack of collaborative registries that would address this shortcoming [39]. In Nepal, we found that suicide data are ‘owned’ by the police force, reinforcing the misperception that it is illegal and categorizing it as a legal issue, rather than a health issue. Such a practice removes an important cause of mortality from the health decision-making, and ultimately from competing for what little resources do exist to address health issues in poor settings. Identifying and resolving principal contradictions among bureaucratic institutions, biomedicine, and culturally congruent understandings of mortality is essential to uncovering the cultural propagation of mortality data. Exploring data ‘transactions’ and bureaucratic categorization allows us to ‘study up’ the issue of suicide, offering opportunities to reveal both the socio-cultural processes by which health statistics get produced and how the discourses used at varying levels of social authority and power shape how death is endorsed and understood [97]. The anthropological community has highlighted concerns related to the governance, oversight, and the impact of high profile public health efforts on state health care systems [98].
4. *Anti*-*stigma efforts are needed to reduce discrimination of persons with self*-*harm behavior and among groups at risk for self*-*harm.* The WHO *Preventing Suicide* report also highlights the need for stigma prevention to improve surveillance, as well as to encourage care-seeking and other preventative measures. In Nepal, prior research has shown that mental health is highly stigmatized through local concepts of “brain-mind” problems (Nepali: *dimaag ko samasya*) [99, 100]. Suicide, similarly, is seen as a brain-mind problem [101]. Campaigns are needed to raise awareness about the causes of suicide, and to combine reporting and seeking services. Raising awareness that suicide is not a crime will also hopefully contribute to reducing stigma. Stigma against suicidal behavior is also prevalent in healthcare settings, as evidenced by these findings in Nepal, as well as through other studies in LMIC [102]. There is a range of models for reducing mental health stigma settings, including reducing stigma associated with self-harm behaviors [103], and these endeavors need to be expanded to develop an evidence base for suicide stigma reduction in LMIC.
5. *Community*-*based detection and reporting should be explored as a complement to institutional surveillance practices.* In Brazil, Nations and colleagues found that community-based surveillance systems produced much more accurate and detailed infant mortality data compared to that of official health statistics [45, 104]. Community-based solutions for detection of public health threats have been successful in Nepal for tuberculosis [105] and for maternal mortality [65]. Pyakurel et al. (2014) specifically called for better detection of suicide cases in Nepal, providing some preliminary evidence that Female Community Health Volunteers (FCHVs) are an ideal candidate for monitoring and reporting deaths. As many suicide deaths remain unreported within the community, using FCHVs as a strategy may improve the lack of police reporting as well as issues related to stigma. Researchers have pointed to the importance of using community informed detection tools for early identification and referral for mental health issues [106]. Training FCHVs in the implementation of such tools alongside a formal collaborative reporting strategy with health and police institutions may drastically improve suicide reporting.

## Conclusions

Death surveillance, and suicide surveillance in particular, remains a fragmented, poorly understood, and disconnected process in Nepal. Results indicate an urgent need for better communication of data systems and their frameworks among those involved in their functioning, particularly health institutions as they remain key stakeholders in the communication and prioritization of health issues. As multi-lateral agencies (UN, WHO, etc.) grow their investment in Nepal, the country is rapidly experiencing health system growth and data systems are the crux of decision making and resource allocation. Further research exploring communication pathways, effectiveness, and the quality of health systems and information systems in resource-poor settings is necessary for a better understanding of what policies and programs are urgently needed to better capture, and ultimately address, hidden health burdens around the world.

## Abbreviations

CIT: Crisis Intervention Team
DfID: Department for International Development
FCHV: Female Community Health Volunteer
GBV: gender Based violence
HMIS: health management information system
LMIC: low and middle income countries
MoFALD: Ministry of Federal Affairs and Local Development
UNICEF: United Nations International Children’s Emergency Fund
USAID: United States Agency for International Development
VDC: Village Development Committee
WHO: World Health Organization
